# Conventional hemodialysis is associated with greater bone loss than nocturnal hemodialysis: a retrospective observational study of a convenience cohort

**DOI:** 10.1186/s40697-016-0118-5

**Published:** 2016-06-01

**Authors:** Bourne L. Auguste, Darren Yuen, Christopher T. Chan

**Affiliations:** Women’s College Hospital, Department of Medicine, University of Toronto, 76 Grenville Avenue, Room 3426, Toronto, ON M5S 1B1 Canada; St. Michael’s Hospital, Division of Nephrology, University of Toronto, Toronto, Canada; Division of Nephrology, Toronto General Hospital, University Health Network, University of Toronto, Toronto, Canada

**Keywords:** Nocturnal home hemodialysis, Conventional hemodialysis, Bone mineral density, DXA, Calcium, Phosphate, Parathyroid hormone

## Abstract

**Background:**

Compared with the general population, end-stage renal disease patients are at increased risk for bone loss and fractures. Nocturnal hemodialysis offers superior calcium-phosphate control and improved uremic clearance compared with conventional hemodialysis. Rates of bone loss by type of hemodialysis are unknown.

**Objectives:**

This study aims to determine whether there are differences in bone loss between frequent nocturnal hemodialysis and conventional hemodialysis.

**Design:**

This is a retrospective observational study.

**Setting:**

Participants were selected from two teaching hospitals in downtown Toronto.

**Participants:**

The study included 88 participants on dialysis for at least 6 months (52 patients on conventional hemodialysis and 36 patients converted from conventional hemodialysis to nocturnal hemodialysis). Patients on peritoneal dialysis and with previous renal transplants were excluded.

**Measurements:**

We obtained demographic variables and biochemical data by a chart review. We examined changes in bone mineral density at the hip (femoral neck, total hip) and spine (L1 to L4) measured at baseline and about 1 year in the two groups.

**Methods:**

We used Student’s *t* test for evaluation of between-group mean differences in demographic and biochemical parameters. We used linear regression models adjusted for baseline age, weight, dialysis vintage, markers of mineral metabolism (serum phosphate, serum calcium, and parathyroid hormone), and baseline bone mineral density at the femoral neck, total hip, and lumbar spine to determine the annualized percent change by hemodialysis type.

**Results:**

Conventional hemodialysis subjects were older than nocturnal hemodialysis subjects (66 ± 9 vs 43 ± 10 years; *p* < 0.0001) with no significant differences in weight, dialysis vintage, serum phosphate, or parathyroid hormone between the two groups at baseline. In a period over 1 year, conventional hemodialysis compared to nocturnal hemodialysis subjects had significantly greater bone mineral density losses at all sites (1.6 % loss at the lumbar spine (95 % confidence interval (CI) 0.2–3.1), 1.3 % loss at the femoral neck (95 % CI 0.1–2.5), and 1.1 % loss at the total hip (95 % CI 0.1–2.6).

**Limitations:**

Some limitations to this study are the lack of medication administration history, short duration (~1 year), and small sample sizes.

**Conclusions:**

This is the first study comparing bone density between hemodialysis modalities. Our study demonstrates that bone loss is less in nocturnal hemodialysis compared to that in conventional hemodialysis which may result in less fractures. Larger observational studies are ultimately needed to confirm preliminary findings from our study.

## Abrégé

**Mise en contexte:**

Les patients qui souffrent d’insuffisance rénale terminale sont plus sujets à la perte de masse osseuse et aux fractures que la population en général. L’hémodialyse nocturne permet une meilleure régulation du phosphate de calcium et améliore la clairance de l’urée par rapport à l’hémodialyse traditionnelle. Néanmoins, le taux de perte de masse osseuse selon le type d’hémodialyse pratiqué demeure indéterminé.

**Objectifs de l’étude:**

Cette étude avait pour but de vérifier s’il existe effectivement des différences dans les taux de perte osseuse selon le type d’hémodialyse pratiqué (hémodialyse nocturne ou hémodialyse traditionnelle).

**Cadre et type d’étude:**

Il s’agit d’une étude rétrospective par observation qui s’est tenue dans deux centres hospitaliers universitaires du centre-ville de Toronto.

**Participants:**

Au total, 88 participants ont été sélectionnés parmi les patients qui recevaient des traitements d’hémodialyse depuis au moins six mois dans ces deux centres hospitaliers. De ce nombre, 52 ont poursuivi leur traitement par hémodialyse traditionnelle alors que les 36 autres sont passés de l’hémodialyse traditionnelle à l’hémodialyse nocturne. Les patients en hémodialyse péritonéale ou ayant subi une transplantation rénale par le passé ont été exclus de l’étude.

**Mesures:**

La consultation des dossiers médicaux a permis d’obtenir les variables démographiques ainsi que les données biochimiques des participants. L’analyse des variations dans la densité minérale osseuse (DMO), mesurée à la hanche (col du fémur et hanche totale) et à la colonne lombaire (L1 à L4), a été effectuée au début de l’étude et environ un an plus tard, pour chacun des groupes de patients.

**Méthodologie:**

Le test T de Student a été utilisé pour l’évaluation des différences de moyennes pour les paramètres démographiques et biochimiques entre les deux groupes de patients. Nous avons employé les mesures initiales de DMO au col du fémur, à la hanche totale et à la colonne lombaire pour déterminer la variation annualisée du pourcentage de perte osseuse. Des modèles de régression linéaire adaptés pour tenir compte des paramètres initiaux des patients tels que l’âge, le poids, l’historique de dialyse et les marqueurs du métabolisme minéral (phosphate sérique, calcium sérique et hormone parathyroïdienne) ont été utilisés pour procéder à l’analyse.

**Résultats:**

Les sujets traités par hémodialyse traditionnelle avaient un âge médian plus élevé que ceux sous hémodialyse nocturne (66 ± 9 ans contre 43 ± 10 ans ; p < 0,000 1). Toutefois, les deux groupes de patients ne présentaient pas de différences significatives au niveau du poids, de l’historique de dialyse, du taux de phosphate sérique ni de l’hormone parathyroïdienne au début de l’étude. Sur une période d’un peu plus d’un an, les sujets sous hémodialyse traditionnelle présentaient des taux de perte osseuse supérieurs pour tous les sites de mesure. La perte osseuse se chiffrait à 1,6 % au niveau de la colonne lombaire (intervalle de confiance [IC] à 95 % entre 0,2 et 3,1), à 1,3 % au col du fémur (IC à 95 % entre 0,1 et 2,5) et à 0,7 % à la hanche totale (IC à 95 % entre 0,1 et 2,6) par rapport aux valeurs mesurées chez les patients pratiquant l’hémodialyse nocturne.

**Limites de l’étude:**

L’absence d’historique sur l’administration de médicaments, la courte durée de l’étude (environ un an) ainsi que la taille restreinte des échantillons de patients limitent la portée de cette étude.

**Conclusions:**

Il s’agit de la première étude à comparer les taux de perte osseuse selon le type d’hémodialyse pratiqué. Notre étude démontre que l’hémodialyse nocturne préserve davantage la DMO que l’hémodialyse traditionnelle, et pourrait incidemment entraîner une réduction du risque de fractures. La réalisation d’études par observation à plus grande échelle serait nécessaire pour confirmer les résultats préliminaires obtenus lors de la présente étude.

## What was known before

There is a paucity of literature on the effects that different modalities of hemodialysis have on bone integrity.

## What this adds

Although this is a relatively small study with some limitations, it provides some preliminary data that suggests that the rate of bone loss is less in a modality of dialysis with better calcium-phosphate balance than frequent nocturnal hemodialysis. Larger follow-up studies would be the key in guiding future research in an area where our understanding of the long-term effects of dialysis on bone homeostasis is limited.

## Background

End-stage renal disease (ESRD) is associated with a three- to fourfold increase in hip fracture risk [1, 2], and fractures are associated with a twofold increased risk of death [3]. Fractures are also related to increased morbidity such that 10 % of patients after hip fracture are functionally disabled and another 19 % require long-term institutionalization in the USA. Fracture-related costs were estimated to be $US 16.9 billion in 2005 and are expected to rise to $US 25.3 billion in 2025 [4]. For example, direct fracture-associated medical costs in a study of American Medicare ESRD patients ranged between $14,475 and $20,810 per fracture event [5].

In otherwise healthy men and women, fracture risk can be evaluated by bone mineral density (BMD) testing using dual energy X-ray absorptiometry (DXA) [6]. In contrast, fracture risk assessment in the ESRD population has been difficult, in part due to underlying metabolic bone disease (MBD). Additionally, the 2009 KDIGO Guidelines does not recommend routine BMD testing because initial cross-sectional studies suggested that BMD did not predict fracture risk as it does in the general population, nor does it predict the type of renal osteodystrophy in patients with CKD stages 3–5 (level 2B evidence) [5].

However, longitudinal data suggests that low BMD predicts fracture, and cross-sectional meta-analytic data confirms that BMD is low in those with CKD and fracture [7–9]. Iimori and colleagues also revealed in a recent longitudinal study that low BMD at the total hip, femoral neck, and distal one third of the radius in stage 5D CKD patients was able to predict fracture risk [7]. KDIGO convened a Controversies Conference in October of 2013 to review the recent CKD-MBD literature since the publication of the 2009 recommendations. The working group believed that recommendations about routine BMD testing in CKD patients should be revisited in future guidelines given data from recent meta-analytic and prospective studies [10].

Furthermore, the rates of bone loss by hemodialysis type are unknown. Home nocturnal hemodialysis (HD) is a novel form of renal replacement therapy that delivers an increased dialysis dose and offers multiple clinical advantages over conventional HD, including the ability to normalize serum phosphate and improve uremic clearance [10–12]. This form of renal replacement therapy occurs while a patient sleeps for five to six nights per week with each session lasting 8–10 h. Given the ability of nocturnal HD to improve phosphate control and uremic clearance, we hypothesized that nocturnal HD use would be associated with less BMD loss than HD over a 1-year time period.

## Methods

### Screening and selection procedures

Our retrospective convenience cohort study included incident and prevalent patients enrolled in the nocturnal HD program of the Toronto General Hospital between 1999 and 2005 and prevalent patients enrolled in the conventional HD program at St. Michael’s Hospital between 1999 and 2005 for more than 6 months. We obtained research and ethics board approval for this study. To be included in our study, patients had to have had two BMD scans separated by at least 8 months and no greater than 14 months apart. In this retrospective convenience sample, we defined study enrollment as the date of the first BMD scan and collected demographic (age at enrollment, gender, weight, cause of ESRD, and dialysis vintage) and biochemical (predialysis plasma-corrected total calcium, phosphate, and parathyroid hormone levels) data. We also recorded biochemical data at 1 year after initial study enrollment. Study patients had BMD scans by a Lunar DXA machine (Lunar Corp, Madison, WI). The patients completed BMD scans for the total hip, the femoral neck, and the lumbar spine in the frontal plane between levels L1 and L4 and were not blinded to dialytic modality. The patients with previous renal transplants and those requiring peritoneal dialytic therapy were excluded from this study.

### Dialysis prescriptions

We performed conventional HD using F80 polysulfone dialyzers (Fresenius Medical Care, Lexington, MA, USA). The dialysate composition was as follows: Na^+^ 140 mM, K^+^ 1–3 mM, Ca^2+^ 1.25–1.5 mM, and HCO3^−^ 40 mM. A blood flow rate (Q_B_) of 400 mL/min and a dialysate flow rate (Q_D_) of 500–750 mL/min were used.

We used either the F80 dialyzers or Polyflux 170 dialyzers (Gambro Inc, Lund, Sweden) for nocturnal HD. The dialysate composition was similar to that used in conventional HD, but often required phosphate supplementation to maintain normal plasma phosphate levels. We used a Q_B_ of 200–300 mL/min and a Q_D_ of 350 mL/min. For both conventional HD and nocturnal HD, vascular access was achieved through a long-term internal jugular catheter, an arterio-venous fistula, or an arterio-venous graft.

### Statistical analysis

We used Student’s *t* test for evaluation of between-group mean differences in demographic and biochemical parameters. Descriptive analyses are presented as mean ± standard deviation. Multiple linear regression models were used to adjust for effects of confounding variables. We identified and adjusted for confounding variables with known risk factors for low BMD and fracture. All statistical tests were performed using the SAS statistical package (SAS). All statistical tests were two tailed, and a *p* value <0.05 was considered significant.

## Results

We identified a total of 88 (52 conventional HD and 36 nocturnal HD) patients in this study (Table 1). Conventional HD patients were older than nocturnal HD subjects (66 ± 9 vs 43 ± 10 years; *p* < 0.0001). We found no significant differences in gender distribution, weight, or dialysis vintage. Although some relevant past medical history was not readily available in some chart reviews, we were able to highlight that there were no major differences in hypertension and vascular disease between the groups from what was available.

**Table 1:** Baseline demographic data for conventional HD and nocturnal HD patients

	Conventional HD	Nocturnal HD
*N*	52	36
Age (years)	66 ± 9	43 ± 10
Cause of ESRD		
Diabetes	32 %	11 %
Glomerulopathy	21 %	36 %
Hypertension	10 %	2 %
PCKD	8 %	14 %
Renovascular	10 %	–
TMA	–	14 %
Unknown	10 %	9 %
Comorbidities^a^		
Diabetes	32 %	11 %
HTN	32 %	28 %
Vascular disease	15 %	17 %
% male	71 %	58 %
Weight (kg)	73 ± 15	73 ± 16
Dialysis vintage (months)	47 ± 30	52 ± 66

Abbreviations: *HTN* hypertension, *PCKD* polycystic kidney disease, *TMA* thrombotic microangiopathy

^a^Comorbidities identified in a chart review for patients at time of enrollment in the study

At baseline, the conventional HD patients had a lower serum calcium level (2.28 ± 0.19 vs 2.42 ± 0.20 mM; *p* = 0.007) compared to the nocturnal HD patients. There were no significant differences in baseline predialysis serum phosphate or parathyroid hormone levels. Serum phosphate levels decreased significantly in the nocturnal HD group from 1.58 ± 0.59 mM to 1.15 ± 0.37 mM (*p* = 0.001), whereas phosphate levels remained elevated and unchanged in the conventional HD group after 1 year. There were no other significant changes in markers of mineral metabolism (Table 2).

**Table 2:** Baseline and 1-year follow-up predialysis biochemical data

	Baseline		1 year	
	Conventional	Nocturnal	Conventional	Nocturnal
Calcium (mM)	2.28 ± 0.19	2.42 ± 0.20†	2.29 ± 0.21	2.42 ± 0.20
Phosphorus (mM)	1.50 ± 0.43	1.58 ± 0.59	1.54 ± 0.42	1.15 ± 0.37*‡
Parathyroid hormone (pM)	42 ± 42	40 ± 52	42 ± 43	42 ± 82

†*p* = 0.007 compared with CHD at baseline

‡*p* < 0.05 compared with CHD at 1 year follow-up

*p < 0.01 compared with NHD at baseline

Predialysis represents lab investigations on the day of dialysis before a dialysis session

### Bone mineral density

The intervals between the bone mineral density (BMD) scans for each group ranged from 8–14 months with means of 11.9 ± 1.2 months and 11.6 ± 0.9 months for nocturnal and conventional HD groups, respectively. The BMD change between the conventional HD and nocturnal HD groups over a 1-year period shows no significant differences at the three measured sites (Table 3). After adjustment for baseline age, weight, dialysis vintage, BMD, and markers of mineral metabolism (total corrected calcium, phosphate, and parathyroid hormone), the rate of decline in BMD was significantly greater in the conventional HD group compared with that in the nocturnal HD group at the total hip, the femoral neck, and the lumbar spine (*p* < 0.05, Table 3).

**Table 3:** Differences in BMD values (g/cm^2^) and % BMD loss over 1 year between the CHD and NHD groups

	Baseline (g/cm^2^)		1 year (g/cm^2^)		% loss (1 year vs baseline)		*p* value BMD after 1 year		% greater BMD loss in conventional HD after 1 year (95 % CI)*	*p* value^a^
BMD site	Conventional	Nocturnal	Conventional	Nocturnal	Conventional	Nocturnal	Conventional	Nocturnal		
L-spine	1.13 ± 0.23	0.93 ± 0.12	1.11 ± 0.27	0.92 ± 0.23	2.5 % (1.2–3.8)	1.0 % (0.1–1.9)	0.685	0.819	1.6† (0.2–3.1)	0.02
Total hip	0.84 ± 0.18	0.92 ± 0.15	0.83 ± 0.25	0.91 ± 0.17	2.1 % (1.1–3.1)	1.0 % (0.2–1.8)	0.815	0.792	1.1† (0.1–2.6)	0.03
Femoral neck	0.77 ± 0.15	0.76 ± 0.14	0.76 ± 0.19	0.75 ± 0.19	2.3 % (1.2–3.2)	1.0 % (0.2–1.8)	0.766	0.800	1.3† (0.1–2.5)	0.02

†All values were statistically significantly lower than NHD patients (*p* < 0.05)

^a^This analysis was adjusted for baseline age, weight, dialysis vintage, bone mineral density, and markers of mineral metabolism (predialysis total corrected calcium, phosphate, and parathyroid hormone)

## Discussion

This study is the first, to our knowledge, to compare the BMD changes between HD and nocturnal HD patients. Our results revealed that bone loss is reduced in nocturnal HD patients over 1 year, possibly conferring lower fracture risk in nocturnal HD. These findings are possibly explained by the lower phosphate levels, reduced calcium-phosphate product, and improved uremic clearance that occurs in nocturnal HD [12–14].

Hyperphosphatemia is a stimulus for parathyroid gland hyperplasia and consequently secondary hyperparathyroidism, causing high turnover bone disease [15, 16]. Several, but not all, reports have suggested an association between high circulating parathyroid hormone levels and increased fracture risk [5, 17]. Furthermore, a recent randomized control trial showed that there was a significant reduction in serum phosphate levels in nocturnal HD patients compared to those in conventional HD [16]. We also observed statistically significant reductions in phosphate levels within the nocturnal HD group after 1 year and compared to our conventional HD group (Table 2). This reduction and even normalization of phosphate may explain the attenuated BMD loss that we observed after 1 year in the nocturnal HD patients.

Nocturnal HD is a novel dialytic modality that offers enhanced uremic clearance through an increase in dialysis frequency and duration. It is well known that larger molecules with increased charge require a longer time to achieve effective clearance due to the selectivity of the dialysis membrane and binding to other proteins. Growing evidence has suggested that phosphate plays an important role as a uremic toxin, possibly potentiating fracture risk [17]. Therefore, it is conceivable that through improved clearance of uremic toxins in nocturnal HD, bone loss and potential fracture risk are reduced.

As it pertains to hyperparathyroidism, our study did not demonstrate any change in mean plasma PTH levels but still demonstrated a significant attenuation in BMD loss in the nocturnal HD cohort. Yuen et al. [11] demonstrated that nocturnal HD lowers parathyroid hormone levels significantly. Walsh and colleagues also showed a reduction in PTH levels in a recent randomized controlled trial but with no significant differences between nocturnal HD and conventional HD patients [14]. The lack of differences in these results including ours may likely be due to dialysis vintage and that modest reductions in phosphate do not alter PTH levels given the progressive nature of moderate and severe secondary hyperparathyroidism. It also supports the belief that other mechanisms may be contributing to bone loss in these patients.

Our unadjusted univariate analysis showed no significant differences in the BMD loss over 1 year between the groups. However, when we adjusted for factors that could contribute to bone loss, conventional HD patients experienced a significantly greater decline in BMD than nocturnal HD patients at the femoral neck, total hip, and lumbar spine as assessed by DXA (Table 3). Although we found no significant differences between PTH levels, we included them in our multivariate analysis as a recent longitudinal study showed HD patients with low or high PTH had higher fracture risk [8].

This study has some limitations. Firstly, it was a retrospective convenience sample and as a result we could not match the nocturnal HD and conventional HD patients for certain confounding variables. We believe that the sampling method may have introduced biases since it was not an inception cohort and a convenience sample in that the study time began when patients had their first BMD scan. These could potentially both introduce biases with similar effects for both types of dialysis and not impacting overall findings.

To address this concern, we used linear regression models to adjust for factors that appear to influence bone fracture risk. We cannot guarantee, however, that our analysis completely adjusted for the effects of these and other potentially unidentified confounders such as physical activity. Additionally, the lack of information about medication administration in this study is a major limitation. Identifying differences in medication administration between the two groups would be important, particularly for agents that may influence BMD such as vitamin D, calcium, or non-calcium-based phosphate binders, cinacalcet, and bisphosphonates.

Despite the lack of differences in PTH values between the two groups, a clearer history and documentation in charts of previous parathyroidectomy surgery would have been useful and could be a potential confounder in our results.

Furthermore, there are likely to be inherent differences between the conventional HD and nocturnal HD populations that can only be corrected for in randomized control trials. For example, we show differences in age and diabetes between the groups representing another significant limitation in this study (Table 1). We did not adjust for diabetes in our multivariate analysis. Recent literature has shown that diabetes is often correlated with increased risk of fracture, but counterintuitively, it is usually characterized by normal or high BMD [18].

Although there were no notable differences in hypertension and vascular disease (Table 1), a more readily available and comprehensive past medical history would have provided useful insight in terms of any significant differences in comorbidities between the two groups.

Ethnicity was not accounted for nor documented in the retrospective data obtained for this study which may have been an additional confounder. A recent systematic review suggested that persons of African descent have higher BMD values at baseline [19]. Although we adjusted for the differences in age between the two groups, it is important to highlight that BMD is well known to decrease with age [20]. Additionally, due to the retrospective nature of this study and because standard clinical protocols only involved assessment of these three sites, we were not able to assess bone density at other relevant sites such as the radius. Recent data has shown that BMD at the radius is useful in predicting fracture risk in ESRD patients [8]. Despite this, quantitative histomorphometry rather than BMD is the preferred method for assessing bone integrity in patients with renal disease [21].

Finally, this study was of short duration (1 year), and involved a small cohort, which in turn limited our statistical power. Consequently, our results may have not fully captured the effects of conventional or home nocturnal HD on bone physiology and strength over a longer period of time.

## Conclusions

Our results in this study suggest that nocturnal HD is associated with less bone loss. We believe that the improved phosphate balance and enhanced uremic clearance observed in nocturnal HD patients plays a pivotal role in attenuated decline in BMD. Although we identified some drawbacks, this is the first study comparing bone density between HD modalities. Given the paucity of literature in this area, conducting larger observational studies along with assessing other variables that may influence BMD are required to confirm our preliminary findings.
